# Methanol leaves extract *Hibiscus micranthus* Linn exhibited antibacterial and wound healing activities

**DOI:** 10.1186/s12906-017-1841-x

**Published:** 2017-06-26

**Authors:** Berhan Begashaw, Bharat Mishra, Asegedech Tsegaw, Zewdneh Shewamene

**Affiliations:** 1 Department of Hospital Pharmacy, University of Gondar Teaching Referral Hospital, Gondar, Ethiopia; 2 Department of Pharmacology, School of Pharmacy, College of Medicine and Health Sciences, University of Gondar, Gondar, Ethiopia

**Keywords:** *Hibiscus micranthus* Linn., Antibacterial activity, Wound healing activity

## Abstract

**Background:**

Infectious diseases are the most common causes of morbidity and mortality in developing countries. Wound and wound infections are also major health problem. Nowadays, medicinal plants play a major role in treatment of infectious diseases and wound healing and they are easily available and more affordable as compared to synthetic compounds. The aim of this study is therefore, to investigate the antibacterial and wound healing activities of 80% methanol extract of *Hibiscus micranthus* leaves using disc diffusion methods and rat excision model respectively.

**Methods:**

In vitro antibacterial screening was carried out against *S. aureus, S.pneumoniae, S. pyogenes, E. coli, P. aeruginosa, K. pneumoniae* and *P. mirabilis* bacterial strains using disc-well diffusion assay. Would healing activity was done in rats divided into four groups each consisting of six animals. Group I was served as a negative control (ointment base), Group II served as a positive control Nitrofurazone (NFZ 0.2% ointment), Groups III and IV was treated 5 and 10% extracts respectively. The acute oral toxicity test and skin sensitivity test were also performed before conducting the actual study. The extract was analyzed for secondary metabolites using standard methods.

**Results:**

Preliminary phytochemical screening have revealed the presence of alkaloids, flavonoids, saponins, tannins, steroids, phenols, diterpines, anthraquinones and the absence of glycosides, terpinoides and triterpines. Based on acute oral toxicity test the extract was found to be safe up to a dose of 2 g/kg. In addition, acute dermal toxicity test indicated no sign of skin irritation. The leaves extract exhibited varying degrees of sensitivity with zones of inhibition ranging from 14.00 ± 0.333 (*S.pyogenes*) to 22.67 ± 1.202 mm (*S.aureus*). It was found that *S. aureus* and *S. pneumonia* (*p* < 0.05) were the most sensitive to the extracts of the leaves at concentrations of 800 μg/ml and 400 μg/ml respectively followed by *P. aeuruginosa* [(18.33 ± .333 mm) (*p* < 0.05)] at a concentration of 400 μg/ml. However, *E. coli* and *P. mirabilis* were found to be resistant to the extract at any of the applied doses. In the wound healing study, the 5 and 10% *w*/w extract exhibited significant wound contraction rate of 99.30% and 99.13% as compared to NFZ ointment and simple ointment base treated groups from 6th to 16th day, respectively (*p* < 0.05).

**Conclusion:**

The present study suggests that the methanol extract of the leaves exhibited a potential antibacterial activity against the tested microorganisms and wound healing activity.

## Background

Infectious diseases are the most common causes of morbidity and mortality in developing countries [1]. Deaths from infectious diseases occur disproportionately in the developing world, where they are the biggest killer of children and young adults [2]. Globally, use of antibiotics has contributed to the dramatic fall in morbidity and mortality from communicable and infectious diseases over the last 50 years. However, the control of infectious diseases is seriously threatened by the steady increase in the number of microorganisms that are resistant to antimicrobial agents [1]. John et al., (2011) described that the widespread use of broad spectrum antibiotics has led to the emergence of several resistant strains of microbes. These contribute significantly towards rise in the escalating health care costs and patients’ morbidity and mortality [3]. In general, bacterial infections are one of the main problems in the world and should be treated by antimicrobial agents. The increased prevalence of known resistant organisms and the emergence of newly resistant organisms have resulted in delayed effective therapy, increase length of hospitalization and have led to increased cost for patients [2].

Wound and wound infections also represent a major health problem both in terms of morbidity and mortality which are most common in developing countries because of poor hygienic conditions [4, 5]. Pattayanac, Sunita (2011) and European Wound Management Association (2005) described that *S. aureus, S. pyogenes, E. coli, P. aeroginosa, S. pneumoniae* and *K. pneumoniae* are some important organisms causing wound infections [4, 5]. Wound infection and associated delayed healing present significant challenges for clinicians predominantly with respect to identifying infection and choosing appropriate treatment options [5].

On the other hand, the world is rich with natural products including medicinal plants. Medicinal plants are now getting more attention than ever because they have potential of numerous benefits as a source of drugs to all mankind. The medicinal value of these plants lies on bioactive phytochemical constituents that produce definite physiological action on the human body. Some of the most important bioactive phytochemical constituents are alkaloids, essential oils, flavonoids, tannins, terpenoids, saponins, phenolic compounds and many more. These natural compounds are the foundations of modern drugs as we know today [6].

**Fig. 1:**
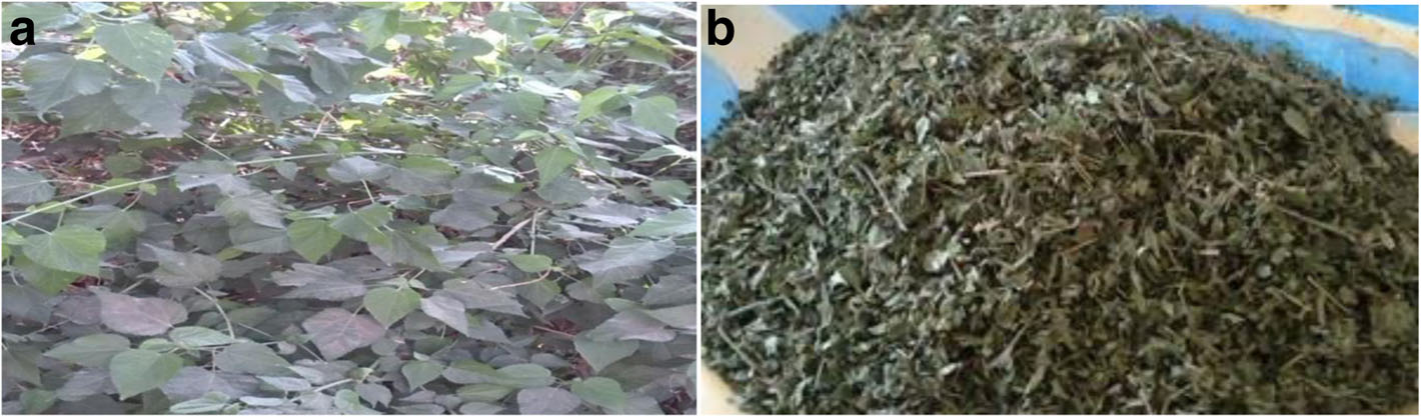
Images of *Hibiscus micranthus* Linn. (**a**) Plant (**b**) Coarse dried powder

***Hibiscus micranthus Linn:*** *Hibiscus is* a genus in the family of malvaceae encompasses more than 300 species [7]*. Hibiscus micranthus* (malvaceae) is a shrub up to 3 m, stem erect, branched, usually with stiff, slender and stellately hairy plant (Fig. 1). There is a constant need for effective and cost-effective therapies to treat bacterial infections and promote wound healing and then plant products offer an alternative that needs to be developed. One alternative will be *H.micranthus*. The research which was done by Naji and Luca (2013), the highest anthocyanin content was obtained from methanol extract of *H. micranthus* than ethanol extract which may be responsible for antioxidant effects [8]. According to the secondary metabolites present in the plant, the plant has been used for its antipyretic, anti-inflammatory, hematological effects, antifungal, antiviral, antitumor, female antifertility, viralizing, as hypoglycemic agent and anabolizing activities [9]. Some compounds like phenolic acids, tannins, flavonoids, β-sitosterol, alkanes, carbohydrates, steroids, fatty alcohols and acids have been reported [10, 11]. The roots are also used traditionally chewed as a cure for cough in India [12], used to cure venereal diseases in Sudan, applied as dressings on wounds and sores of humans and domestic animals and are also taken to cure bronchitis and pneumonia in Kenya and Tanzania [13]. In Tanzania, the leaves are used for treating earache, the leaves sap is taken against dysentery, water in which leaves have been pounded is taken against stomach-ache and leaf pulp is applied on swellings, used as an antidote for snakebites and as a treatment for kidney problems. In Tanzania and Zambia, the whole plant is used to treat convulsive fever in children [13].

In Ethiopia, the leaves of this plant provides medicines traditionally for treating skin burning (dermatological infections) and skeleto muscular disorders [8], swellings over body [14, 15], for wound healing or sore in Tigray region [16]. The leaf and the flower of the plant is also used for wound and dermatological purposes by chewed and creamed with cotton in Amhara region, in and around Tara Gedam, South Gonder Zone [17]. *Hibiscus micranthus* also uses for burn in Negelle-Borona, Ethiopia [18].

Sohel A. et al., demonstrated that the flower extracts of *Hibiscus rosa-sinensis* had stronger antibacterial effects [19]. Shivanada et al., has also found that an ethanol extract of *Hibiscus rosa sinensis* has properties that provide it capable of promoting accelerated wound healing activity in animal model [20]. From literature search, no scientific investigations have been conducted till date to verify the use of *H. micranthus* as antimicrobial and wound healing remedies even though the *Hibiscus rosa sinusis* has approved for wound healing. That is why the present study, therefore, focuses on delineation of the antibacterial activities of extracts of *H. micranthus* and its effects on wound by excision wound model. Many plant materials including *H. micranthus* have been used topically and systemically to enhance wound repair though evidence is lacking. There is, however, a need to study and provide evidence for the efficacy of *H. micranthus* extract in the treatment of wounds. The main compounds of the plant such as alkaloids, flavonoids, tannins, steroids, saponins, phenols, diterpines and anthraquinones will support biological effects. The results of pharmacological screening of *H. micranthus* will have a contribution in the discovery of various medicinal plants with antibacterial and wound healing activities.

## Methods

### Chemicals and reagents

Ampicillin, ciprofloxacin, methanol (RFCL limited, New Delhi, India), nutrient broth (India), blood agar, MacConkey agar, Mueller Hinton Agar, biochemical testing, DMSO (dimethyl sulphoxide- Loba Chemie Pvt.Ltd. Mumbai, India), phytochemical screening reagents, ketamine (ROTEXMEDICA, Germany), simple ointment base and nitrofurazone ointment USP 0.2% (Galentic pharma, India Pvt. Ltd.) were purchased and collected from the respective sources.

### Plant material collection and identification

Enough amounts of the fresh leaves of *H. micranthus* were collected from Gondar area, Ethiopia during the Month of January 2015. The plant was identified by a Taxonomist (Mr. Melaku Wondaferash) and a voucher specimen representing *H. micranthus* (specimen No. AA 004) was deposited at the National Herbarium, Department of Biology, Addis Ababa University, Ethiopia.

### Test organisms

In the present study, the gram positive bacterial strains used were *S. aureus* (ATCC2923), *S. pneumoniae* (ATCC137348) and *S. pyogenes* (ATCC19615) while gram negative bacteria strains used were *E. coli* (ATCC1925525), *P.aeruginosa* (ATCC27853), *K.pneumoniae* (ATCC70060) and *P. mirabilis* (ATCC12386) obtained from the American Type Culture Collection (ATCC) to determine the antibacterial activity of *H. micranthus*. The bacterial strains were obtained from University of Gondar Referral Teaching Hospital Laboratory and Microbiology Department. Pure culture of bacteria was maintained at 4 °C on nutrient agar slants. The identity of these bacterial isolates was confirmed through the conventional biochemical tests [19, 21].

### Experimental animals

Healthy adult Wistar albino rats of either sex were selected randomly for the study. Rats were obtained from animal breeding house of School of Pharmacy, University of Gondar. Rats of 12 weeks, weighing 160–220 g were used for experiment. Each rat was housed in 2200cm^2^ polypropylene cage floor area under controlled conditions at 19-25 °C and kept under 12/12 light/dark cycle with free access to standard pellet feed brought from Ethiopian Health and Nutrition Research Institute, Addis Ababa and water ad libitum*.* They were all acclimatized to the Pharmacology Laboratory prior to use. The study was carried out according to the National Research Council Guide for the Care and Use of Laboratory Animals and Organization of Economic Co-operation and Development (OECD) guide line [22, 23] after obtaining the approval of the Institutional Animal Ethical Committee (IAEC). Six animals in each group and duration of observation required to obtain consistent data were employed.

### Experimental design

The study utilized an in vitro antibacterial and in vivo experimental study for acute oral and dermal toxicity test and for wound healing activity test. Crude extract of the plant leaves was prepared with maceration. The extract was then screened for phytochemical, acute oral and dermal toxicity tests, antibacterial and wound healing activity tests by their respective methods.

### Extraction procedure

Fresh matured leaves of *H. micranthus* was collected, washed, dried under shade and extracted as described by Kumar et al.*,* [11] with slight modification. The dried leaves were coarsely powdered using a mortar. Then, this coarsely powdered plant was macerated in 80% methanol to obtain the hydro-alcoholic crude extract using Erlenmeyer flask for 3 days at room temperature. After 72 h, the filtrate was separated from the marc by using filter paper (Whatman No.1). The marc was re-macerated twice. Then the alcohol was allowed to evaporate from the filtrate with mild heating on dry oven to dry at 40 °C and then the concentrated extract was stored at 4 °C until the actual experiment is done.

### Preliminary phytochemical screening

The crude methanol extract was assessed for secondary metabolites such as phenol, tannin, flavonoids, saponins, terpenoids, triterpenes, diterpines, glycosides, anthraquinones, alkaloids and steroids using standard methods [6, 11, 20].

### Acute oral toxicity test

Acute toxicity study was carried out using the limit test dose of 2 g/kg as described by OECD (2008) guideline −425 and Interagency Research Animal Committee Recommendation [22, 23]. Five animals of either sex were fasted overnight but water was allowed and was administered with the limit dose 2 g/kg of *H. micranthus* extract. Animals were observed individually for behavioral profile (alertness, restlessness, irritability and fearfulness), autonomic profiles (defecation and urination), neurologic profile (spontaneous activity, reactivity, touch response, pain response and gait), physical states as lacrimation, loss of appetite, tremors, hair erection, salivation, diarrhea and for mortality after dosing at attentively during the first 30 min, with special attention given during the first 4 h, periodically during the first 24 h and daily thereafter for a total of 14 days.

### Acute dermal toxicity test

The skin irritation test with 80% methanol extract of *Hibiscus micranthus* was conducted on rats. Animals showing normal skin texture were housed in a cage. Five rats were employed for this test and their skin was shaved on the dorsal side, each about 400 mm2 areas 24 h before application of the sample. A limit test dose of 2000 mg/kg of formulation was applied uniformly over the shaved area for 24 h. The plant extract was held in contact with the skin using a zinc oxide adhesive plaster and a non-occlusive bandage dressing for 24 h. At the end of the exposure period, the bandage and the test materials were removed and observed for an adverse skin reaction/irritation. Then, they were further observed for 14 days for any signs of toxicity [24–27].

### Methodology for detection of antibacterial activity

#### Inoculums preparation

The bacterial isolates were first grown in a nutrient broth for 18 h before use. The microbial inoculums were standardized by 0.5 McFarland. Inoculums preparation of 0.5 McFarland turbidity standards was done by using a sterile inoculating loop touch isolated colonies of the same morphology were suspended in 5 ml of nutrient broth in to sterile test tubes for the identified bacteria. 0.5 McFarland standards were vigorously agitated to turbidity on a vortex mixer before use. As with the barium sulfate standards, a 0.5 McFarland standard was comparable to a bacterial suspension of 10^8^ cells per/ml. The standard was compared visually to a suspension of bacteria in nutrient broth accordingly [21].

#### Antibacterial assay

The antibacterial activity of methanolic extracts of *H. micranthus* leaves was tested against clinical isolates by agar-well diffusion method as described by Sohelet al., and Bahur et al., [19, 27]. A Cork borer of 6 mm diameter was used to punch well in agar plates to cut uniform wells [19]. Then, an aliquot of 100 μl inocula for each bacterial isolate was evenly spread by a sterile glass spreader onto a previously bored on to Muller Hinton Agar using sterilized cotton swab and was allowed at room temperature. Different concentrations of the extract (200, 400 and 800 μg/ml) were prepared using DMSO as solvent. Subsequently, 200, 400 and 800 μg/ml extracts of leaves were poured into the wells. Ciprofloxacin 5 μg and ampicillin 10 μg discs were used as positive control. DMSO was used as a negative control. Then the plates were kept at 2-8 °C in a refrigerator to allow diffusion of the extracts in to the agar and further incubated at 37 °C for 24 h. The diameter of zone of inhibition was measured to the nearest millimeter [19, 28]. The formation of clear inhibition zone of ≥7 mm diameters around the wells was regarded as significant susceptibility of the organisms to the extract [29]. The effect was compared to those of antibiotic discs. The tests were performed in duplicates and the mean was taken. The whole experiments were performed under strict aseptic conditions.

### Determination of Minimum inhibitory concentration (MIC) and Minimum bactericidal concentration (MBC)

The MIC test was performed for bacteria species susceptible by disc diffusion test and the estimation of MICs of the extraction was investigated per the Clinical Laboratory Standards Institute [30]. MIC was conducted using macro-tube serial dilution methods. The extract (stock) solution was prepared and serially dilutions were done in DMSO. The extract solutions were prepared in different test tubes. The extract solution (5 mg/ml) was serially diluted as 1:1, 1:2, 1:4, 1:8 and 1:16 to bring 5 mg/ml, 2.5 mg/ml, 1.25 mg/ml, 0.625 mg/ml and 0.3125 mg/ml concentrations respectively. Varying concentration of the extract (5 mg/ml, 2.5 mg/ml, 1.25 mg/ml, 0.625 mg/ml and 0.3125 mg/ml) in test tubes containing 10 ml nutrient broth was added and then a loopful of the test organisms was introduced in each test tube. All inoculated dilutions were incubated at 37^O^c for 24 h. The lowest concentration of the plant extract that retains its inhibitory effect resulting in no growth (absence of turbidity) of a microorganism was recorded as the MIC value of the extract.

Determination of MBC of the extract was also investigated by the method described by the Clinical Laboratory Standards Institute [30]. A loopful sample was then taken from each set of test tubes with no visible growth in MIC assay and sub-cultured on freshly prepared Nutrient agar plate and later incubated at 37^O^_C_ for 24 h. After incubation, the lowest concentration of the plant extracts showing no bacteria growth was recorded as MBC.

### Grouping and dosing of animals

The animals were randomly assigned to four groups of each consisting of six rats each. The animals of Group I was used for simple ointment base, Group II was served as reference standard and treated with 0.2% *w*/w nitrofurazone ointment and animals of Group III and IV were treated with ointments prepared from 5% and 10% concentrations of methanolic extract of *H. micranthus* respectively*.* Dose of ketamine anaesthesia (50 mg/kg, ip) for wounding procedure was selected based on previous studies120mg/kg, ip [31], 30 mg/kg,ip [32] and 50 mg/kg sc [25]. The test doses were prepared freshly on the day of the experiment.

### Methods for wound healing activity

#### Ointment formulation

Two types of ointment formulations were prepared from the extract. Five percent (*w*/w) and 10% (*w*/w), where 5 or 10 g of the extract were incorporated in to 100 g of simple ointment base respectively [33, 34]. Nitrofurazone ointment (0.2% *w*/w) was used as a standard drug for comparing the wound healing potential of the extract [31, 34].

#### Methods for creating excision wound

The animals were anesthetized with ketamine 50 mg/kg body weight. The rats were inflicted with excision wounds as described by Mekonnen *et al*., and Morton and Malone [25, 35]. The dorsal fur of the animals was shaved with small scissors and the anticipated area of the wound to be created was outlined on the back of the animal with marker. The excision wound was made by cutting away a circular area 210–240 mm^2^ and 1–2 mm depth full thickness of skin from the depilated area along the marking using toothed forceps, a surgical blade and pointed scissors. The wound was left undressed to open environment [25, 31].

#### Treatment methods

The ointment was topically applied once a day starting from the day of the operation till complete epithelization. This model was used to monitor wound contraction and wound closure time. Wound contraction was calculated as percentage reduction in wound area. The progressive changes in wound area were monitored planimetrically by measuring the diameter every alternate day. The period of epithelialization was calculated as the number of days required for falling of the dead tissue remnants of the wound without any residual raw wound [31].

The wound area was measured on alternate days and the epithelialization period recorded at the end of the study. Wound contraction (%) was calculated using the relation [36, 37]:

$$ \mathrm{Wound}\ \mathrm{contraction}\ \left(\%\right)=\left[\left(\mathrm{WA}0-\mathrm{WAt}\right)/\mathrm{WA}0\right]\ 100 $$

Where:

$$ \mathrm{WA}0=\mathrm{the}\ \mathrm{wound}\ \mathrm{area}\ \mathrm{on}\ \mathrm{day}\ \mathrm{zero} $$

$$ \mathrm{WAt}=\mathrm{the}\ \mathrm{wound}\ \mathrm{area}\ \mathrm{on}\ \mathrm{day}\ \mathrm{t}. $$

### Data analysis

Statistical analysis was performed using one-way analysis of variance (ANOVA) with post hoc Tukey’s multiple range tests with SPSS version-16 for windows. *P* < 0.05 was considered significant and all data was expressed as mean ± SEM.

### Ethical considerations

Animal handling was done according to the guide lines for Care and Use of Laboratory Animals [22] and OECD-guidelines-425 [23]. The proposal was submitted to Department of Pharmacology for approval and then the study was undertaken after obtaining the approval of IAEC of University of Gondar (IAEC approval letter No. SoP4/407/2015 dated 28th April 2015). The certificate of clearance was obtained from Department of Pharmacology to proceed the research.

## Results

### Yield of the plant’s extract

The *Hibiscus micranthus* leaves were extracted for the crude active extracts of 80% methanol. From 100 g of *H. micranthus* leaves extract, 11.6%*w*/w was extracted using methanol. The extract was greenish black.

### Phytochemical screenenig

Chemical analysis of the powdered plant was carried out qualitatively to associate the antibacterial and wound healing activities of the plant extract. Preliminary phytochemical screening of the leaves extract of *H. micranthus* revealed the presence of alkaloids, flavonoids, saponins, tannins, steroids, phenols, diterpines, anthraquinones and the absence of terpenoids, glycosides and triterpines (Table 1).

**Table 1:** Phytochemical profile of methanol extract of *Hibiscus micranthus* leaves

Chemical constituents	Inference
Alkaloids	+
Flavonoids	+
Saponins	+
Tannins	+
Steroids	+
Phenols	+
Diterpines	+
Anthraquinones	+
Terpenoids	-
Glycosides	-
Triterpines	-

+ = present, − = absent

### Antibacterial activity of plant extracts using agar well diffusion

The extract showed antibacterial activity as indicated by the zone of growth inhibition ranged from 14 ± .000–22.67 ± 1.202 mm. *S. aureus (*which show significant difference with the positive control ampicillin at *p = 0.021* and *S. pneumoniae* strain in a concentration dependent fashion (which shows significant difference with the positive control ampicillin at *p = 0.003* and ciprofloxacin at *p = 0.016)* had the largest zone of inhibition (22.67 ± 1.202 mm) at concentration of 800 μg/ml and 400 μg/ml respectively followed by *P.aeuruginosa*[(which shows significant difference with the positive control ampicillin at *p = 0.037* and ciprofloxacin at *p < 0.001)* (18.33 ± .333 mm)] and *K.pneumoniae*[(which shows significant difference with the positive control ampicillin at *p = 0.040* (16.33 ± 1.202 mm)] at a concentration of 400 μg/ml and 800 μg/ml respectively while *S.pyogenes* had the smallest zone of inhibition (14 ± .000 mm) at a concentration of 400 μg/ml. However*, E.coli* and *P.mirabilis* were found to be resistant (.00 ± .000 mm) to the leaves extract at any of the applied doses (Table 2).

**Table 2:** Diameter of zone of inhibition (mm) against bacteria by *H.micranthus* leaves extract

Bacterial strain	Conc.μg/ml	*p*-value	Between	Mean zone of inhibition(mm) ± SEM			
				Extract	Cipr 5 μg	Ampi 10 μg	DMSO100μl
*S. aureus*	200	0.021	Extract-amp	18.33 ± 1.667	21.67 ± 1.202	25.33 ± 1.667	.00 ± .000
	400	-	-	20.00 ± 1.555			
	800	-	-	22.67 ± 1.202			
*S.pneumoniae*	200	-	-	20.67 ± 1.764	19.33 ± .333	21.00 ± .577	.00 ± .000
	400	-	-	22.67 ± 1.202			
	800	0.016 0.03	Extract-cipr Extract-amp	13.00 ± 1.528			
*S.pyogenes*	200	-	-	.00 ± .000	23.33 ± .667	27.00 ± .577	.00 ± .000
	400	-	-	14 ± .000			
	800	-	-	.00 ± .000			
*P.aeuruginosa*	200	<0.001	Extract-cipr	.00 ± .000	27.00 ± .577	6.00 ± 6.00	.00 ± .000
	400	0.037	Extract-amp	18.33 ± .333			
	800	<0.001	Extract-cipr	.00 ± .000			
*K.pneumoniae*	200	0.013	Extract-amp	13.67 ± 1.856	18.00 ± 1.453	20.00 ± .577	.00 ± .000
	400	0.040	Extract-amp	14.67 ± .667			
	800	-	-	16.33 ± 1.202			
*E.coli*	200	-	-	.00 ± .000	23.67 ± 1.453	19.33 ± .333	.00 ± .000
	400	-	-	.00 ± .000			
	800	-	-	.00 ± .000			
*P.mirabilis*	200	-	-	.00 ± .000	30.00 ± .000	26.33 ± .333	.00 ± .000
	400	-	-	.00 ± .000			
	800	-	-	.00 ± .000			

Conc- concentration of the extract; Cipr-ciprofloxacin; Amp -ampicillin; DMSO–Dimethyl sulphoxide, mm-millimeter, μg-microgram, μl-microlitre; ml-mililitre; SEM-standard error of the mean; ‘0’ - no zone of inhibition;(−) - no significant difference between extract and standard drugs. *P*-values <0.05 indicates significant difference between the extract and the standard drugs or positive controls. Values greater than 6 mm diameter of the well indicate some activity

### Minimum inhibitory concentration (MIC) and Minimum bactericidal concentration (MBC)

MIC and MBC were tested for the methanol extract of *H. micranthus* leaves and the results are presented in Table 4. *S. pneumoniae* had the lowest MIC value (0.625 mg/ml), while *S. aureus* and *P.aeuruginosa* had MIC value of 2.5 mg/ml and 1.25 mg/ml respectively. *K.pneumoniae* had the highest MIC value of 5 mg/ml. Higher MBC of 5 mg/ml was observed against *S. aureus*, *P. aeuruginosa* and *K.pneumoniae.* MBC value of 1.25 mg/ml against *S. pneumoniae* was reported (Table 3).

**Table 3:** MIC and MBC of *H.micranthus* leaves extract against test organisms

Bacterial strain	MIC (mg/ml)	MBC (mg/ml)
*S. aureus*	2.5	5
*S. pneumonia*	0.625	1.25
*S.pyogenes*	ND	ND
*P.aeuruginosa*	1.25	5
*K.pneumoniae*	5	5
*E.coli*	ND	ND
*P.mirabilis*	ND	ND

*ND* Not Determined, *MIC* Minimum Inhibitory Concentration, *MBC* Minimum Bactericidal Concentration, *mg/ml* milligram per milliliter

### Results of acute oral toxicity

All the rats which received *H. micranthus* at the dose up to 2 g/kg body weight did not result in signs of toxicity or mortality. The animals were physically active, consuming food and water as regular. Any sign of abnormal behavior has not been noticed. Based on the result of acute toxicity, LD50% was estimated to be above 2 g/kg body weight.

### Results of acute dermal toxicity

The purpose of this study was to assess the skin irritation potential of *Hibiscus micranthus* from a single topical application. During the entire period of experimentation, no animal showed a sign of skin and fur change, edema, salivation, diarrhoea and any other behavioral pattern.

### Wound healing effect of *Hibiscus micranthus* in excision wound model

The measurements of the progress of the wound healing include by the NFZ ointment (0.2%*w*/w), extract ointment 5 and 10% *w*/w and the control group (simple ointment base) in the excision wound model are shown in Table 4. The 5% and 10% *w*/w contained 5 g and 10 g of the extract in 100 g of simple ointment base, respectively. The two doses of the plant extract 5 and 10% *w*/w exhibited significant wound contraction rate as compared to NFZ ointment and simple ointment base treated group from 6th to 16th day respectively (*p* < 0.05). It was found that the wound healing contracting ability of the extract ointment in different concentrations was significantly greater (*p* < 0.05) than that of the control (i.e. simple ointment treated group) as well as reference standard (NFZ ointment) starting from the sixth day onwards. The extract ointment produced complete healing at the 16th day when 5 and 10% *w*/w extract ointments were used. The extract treated wounds were found to epithelialize faster compared to the control group.

**Table 4:** Effect of the methanol extract of *H.micranthus* leaves on excision wound model (*n* = 6)

Post wounding days	Wound surface area (mean ± SEM) in mm^2^ and percentage of wound contraction			
	Ointment base	Nitrofurazone ointment (0.2%*w*/w)	Extract ointment (5% *w*/w each)	Extract ointment (10% *w*/w each)
0	220.60 ± 2.713 (0.0)	213.80 ± 7.971 (0.0)	227.80 ± 5.643 (0.0)	230.80 ± 2.871 (0.0)
2	180.80 ± 4.769 (18.04)	161.20 ± 7.010 (24.60)	166.00 ± 9.508 (27.13)	167.80 ± 12.395 (27.30)
4	145.40 ± 7.096 (34.09)	136.00 ± 6.782 (36.39)	119.20 ± 10.851 (47.67)	117.40 ± 9.765 (49.13)
6	113.80 ± 5.643 (48.41)	80.00 ± 8.390 (62.58)	76.20 ± 8.777* (66.55)	83.40 ± 10.028 (63.86)
8	70.80 ± 11.128 (67.91)	44.40 ± 4.261 (79.23)	36.60 ± 5.600* (83.93)	53.60 ± 7.467 (76.78)
10	34.60 ± 4.707 (84.32)	20.60 ± 2.088 (90.36)	16.80 ± 1.497* (92.63)	29.40 ± 5.400 (87.26)
12	19.80 ± 1.428 (91.02)	14.40 ± 2.400 (93.26)	10.80 ± 1.200* (95.26)	11.60 ± 2.400* (94.97)
14	9.40 ± 1.166 (95.74)	7.20 ± 3.200 (96.63)	2.40 ± 0.400 (98.95)	5.20 ± 1.068 (97.75)
16	6.60 ± 0.872 (97.00)	3.80 ± 2.835 (98.22)	1.60 ± 1.666 (99.30)	2.00 ± 1.095 (99.13)

Values in parenthesis indicate percentage of wound contraction.* significant differences at *p* < 0.05 (statistical analysis was done by one-way analysis of variance (ANOVA) followed by Tukey’s test for multiple comparisons)

## Discussion

Now a day, medicinal plants play a major role in the treatment of infectious disease [19, 38–42]. The World Health Organization (WHO) estimates that plant extract or their active constituents are used as folk medicine in traditional therapies of 80% of the words population. Ethnobotanical investigations have been found to recommend main clues in the discovery and development of traditionally used medicinal plants into modern drugs [43]. The medicinal values of these plants lie in bioactive phytochemical constituents that produce definite physiological function on the human body and those compounds formed are the foundations of modern prescription drugs as we know today [6].

Antibiotic resistance is a major concern and development of new agents from plants could be useful in meeting the demand for new antimicrobial agents with improved safety and efficacy.

Newer antimicrobial agents from plant extracts may be useful in food, dairy and pharmaceutical industries to prevent contamination by limiting the microbial growth [44]. The leaves extract of *H. micranthus* demonstrated significant antibacterial activity against most of the test organisms. The extract was more potent against gram-positive *S. aureus* and *S. pneumoniae* with maximum zone of growth inhibition of 22.67 ± 1.202 mm at 800 μg/ml and 400 μg/ml respectively. Though, *S. pyogenes* had the smallest zone of inhibition (14 ± .000 mm) compared with other gram positive bacteria at a concentration of 400 μg/ml, it was found that the extract had an inhibitory effect on this strain.

The plant extract inhibited gram-positive microorganisms better than the gram-negative ones. This is in agreement with previous reports [45]. Although gram-negative bacteria tend to have higher intrinsic resistance to most antimicrobial agents [46], impressive activity against some gram negative bacteria was observed. Low values of MIC (0.625 mg/ml) and MBC (1.25 mg/ml) demonstrated by the extract especially on *S. pneumonia*e is an indication that the phytoconstituents have bactericidal potential. Similarly, the MIC (1.25 mg/ml) and MBC (5 mg/ml) exhibited against *P. aeuruginosa.* The inhibitory effects of the extract of *H. micranthus* leaves against pathogenic bacterial strains can introduce the plant as potential candidate for drug development for treatment of ailments caused by these pathogens.

The results of the antibacterial effect of *H. micranthus* on *E.coli* and *P. mirabilis* indicate that these bacteria present resistance even in higher concentration the fact that the diameter of the inhibition zone was zero in all petridishes inoculated by these strains. While the antibacterial activities of the methanol extract of *H. micranthus* leaves compared to standard antibiotics (ciprofloxacin and ampicillin) showed broad spectrum as its activity were independent on gram reaction. The extract tested showed antimicrobial activity against both gram positive (*S.aureus, S.pneumoniae* and *S.pyogenes*) and gram negative organisms (*P.aeruginosa* and *K.pneumoniae*). The Present investigation confirms, therefore, the antibacterial activity of extracts of *H. micranthus* leaves. These activities are supported by the presence of high level of alkaloids, diterpines, tannins, saponins, phenols, flavonoids and steroids which might be responsible for the antibacterial activities [6, 46]. Anthraquinone also has antibacterial effect as described by Marjorie Murphy Cowan [47].

In the phytochemical screening of this study, the methanol extract of *H. micranthus* showed positive indication for the presence of alkaloids, flavonoids, saponins, tannins, steroids, phenols, diterpines and anthraquinones. Therefore, the observed antibacterial activity of the methanol extract can be attributed primarily by the nature of biologically active components of the plant like alkaloids, diterpines, tannins, saponins, phenols, flavonoids and steroids which are well known for their antimicrobial activity [6, 44] supporting the traditional use of the plant and secondarily by the stronger extraction capacity of methanol could have produced a large number of active constituents responsible for antibacterial activity [8, 42]. Expected mode of antimicrobial action of some secondary metabolites may be related for example tannins inactivate microbial adhesins, enzymes and cell envelope transport proteins; flavonoids targeted on microbial membrane; alkaloids intercalate in to cell wall and /or Deoxyribonucleic acid (DNA) where as diterpines and phenolic substances disrupt microbial membrane [47]. These secondary compounds may come into play either individually or synergistically to confer the antibacterial potential of this plant.

From the literature, the n-hexane extract of the *H. micranthus* leaves contains aldehydes that has been used for antiseptic and also contains phenolics like eugenol usually posses antioxidant, antimicrobial, antifungal and used as several other therapeutic uses [10]. The highest anthocyanin content of methanol extract of *H. micranthus* may be responsible for antioxidant effects [8].

These were in support of the current study in having antibacterial and wound healing effect. Findings of the present study clearly demonstrate the scientific basis of traditional medication with the extracts prepared from *H. micranthus* and reveals its potential use as complementary and alternative medicine.

Wound healing process begins with restoration of damaged tissue as closely as possible to its natural state and wound contraction is the course of shrinkage in wounded area [48]. In the present study, topical application of *H. micranthus* leaves extract on excision wound showed increased rate of wound contraction and reduced epithelialization period in rats. Thus, the effect of the extract on wound contraction and epithelialization suggests, it may enhance epithelial cells migration and proliferation as well as the formation, migration and action of myofibroblasts. The extract may also stimulate processes associated with tissue regeneration. The significant responses to the extract reported above may be attributed to properties that include antioxidant, anticonvulsant, and hypoglycemic activities [31]. A variety of in vitro and in vivo experiments have shown that some plants metabolites such as tannins, phenolic compounds, flavonoids and terpenoids promote wound healing process mainly due to their astringent and antimicrobial property which seems to be responsible for wound contraction and increased rate of epithelialization [49]. Flavonoids, tannins and simple phenolic compounds possess anti-inflammatory and antioxidant properties and could contribute to the wound healing properties of *H. micranthus*. Flavonoids are known to reduce lipid per oxidation by preventing or slowing onset of cell necrosis and improving vascularity, hence increasing the strength of collagen fibers by increasing circulation or by preventing cell damage and by promoting DNA synthesis [49]. Previous studies also showed that the chemical constituents of *H. micranthus* contained the anthocyanin which may be responsible for antioxidant effects [8]. The extract showed positive indication for the presence of alkaloids, flavonoids, saponins, tannins, phenols, diterpines and anthraquinones which are responsible and supported the wound healing activity. These bioactive agents usually modulate one or more phases of the healing process and are multifunctional natural products that act through multiple targets by being anti-inflammatory, antioxidant, etc.

Alkaloids promote early phases of wound healing (≤ 7 days), stimulate the growth of colonies from fibroblast precursors. On the other hand, saponins have been shown to modulate wound cells function which can increase both fibroblast proliferation and migration, showing greater cell density, more regularly organized dermis and more newly formed blood vessels.

The anthraquinone has been shown to promote excisional wound repair in rats via complex mechanism involving stimulation of tissue regeneration. Tannins typically act as astringents which are responsible for wound contraction and increased rate of epithelialization at the granulation formation and scar remolding phases. The wound healing effect of tannins may also be attributed to their anti-inflammatory activity due to their antioxidant action [50]. Phenolic compounds have been documented to possess potent antioxidant and free radical scavenging effect, which is believed to be one of the most important components of wound healing. Sulfur containing compounds are also important in healing accelerated efficacy and antimicrobial potency [34]. The extract has been reported to have a broad spectrum antibacterial activity in this study, which also seemed to have beneficial effects on promoting wound healing.

The result showed that the wound area recorded for each extract-ointment decreased as the days of exposure increased, while the respective percentage wound contraction increased with increase in the number of days of exposure to the extract ointments. It was observed that the wound healing contracting ability of the extract ointment in different concentrations was significantly greater (*p* < 0.05) than that of the control (i.e. simple ointment treated group) as well as reference standard (NFZ ointment). The 5% (*w*/w) extract ointment treated groups showed significant wound healing (*p* < 0.05) from the 6th day onwards where as the 10% (*w*/w) extract ointment treated group showed significant wound healing (*p* < 0.05) from the 12th day. The percentage of wound contraction was somewhat much more with the 5% *w*/w extract ointment treated group (1.60 ± 1.666 mm^2^) than the 10% *w*/w extract ointment treated group (2.00 ± 1.095 mm^2^) and the NFZ ointment (3.80 ± 2.833 mm^2^) area on the 16th day respectively. The wound healing activity of the plant extract may also be due to its angiogenic and mitogenic potential leading to increased cellular proliferation and increased collagen synthesis. Because of these properties, *H. micranthus* has been used widely in herbal medicines for burns, psoriasis and prevention of scar formation following surgery [6].

It appears that *H. micranthus* has prohealing effect as evidenced by the above findings and was able to promote epithelialization either by facilitating the proliferation of epithelial cells or by increasing the viability of epithelial cells. Due to the various properties as discussed above, *H. micranthus* could be used to treat open wounds; however, confirmation of this conclusion requires clinical evaluation.

## Conclusion

The present study suggests that methanolic leaf extract of *H. micranthus* has a potential antibacterial activity against the tested microoragnisms and promoted a rapid would healing activity. These effects would be attributed to the presence of phytoconstituents like alkaloids, flavonoids, saponins, tannins, steroids, phenols, anthraquinones and diterpines. The extract was safe and shows no sign of toxicity or mortality with a dose of 2 g/kg body weight.

## Abbreviations

ANOVA: Analysis of variance
DMSO: Dimethyl sulphoxide
DNA: Deoxyribonucleic acid
*H. micranthus*: *Hibiscus micranthus*
NFZ: Nitrofurazone
